# Supplementary material for: Priming of Colorectal Tumor-Associated Fibroblasts with Zoledronic Acid Conjugated to the Anti-Epidermal Growth Factor Receptor Antibody Cetuximab Elicits Anti-Tumor Vδ2 T Lymphocytes
Source: Cancers (Basel). 2023 Jan 18;15(3):610. doi: 10.3390/cancers15030610 (PMC9913507; doi:10.3390/cancers15030610)
Supplement: Supplementary file 1 [file cancers-15-00610-s001.zip › cancers-2138914-supplementary.pdf]

Table S1. Pathology features of CRC specimens used in this study to generate TAF primary cell lines.

CRC sample ID	Age	Gender	Site	Stage UICC	T	N	M	G	Microsatellite instability	Mucinous
**OMCR15-061TK**	67	F	S	IIA	3	0		2		
**OMCR15-062TK**	52	M	S	IVA	3	2b	1a	3		
**OMCR15-063TK**	73	F	R	IIC	b	0		3	MSI	
**OMCR15-066TK**	78	F	L	IIA	3	0		2		
**OMCR15-072TK**	76	M	R	IIA	3	0		2		
**OMCR15-073TK**	82	F	R	I	2	0		2		<50%
**OMCR15-078TK**	53	F	L	IIA	3	0		2		
**OMCR15-082TK**	73	M	R	IIA	3	0		2		<50%
**OMCR16-001TK**	68	M	RT	IIIC	3	2b		2		
**OMCR16-004TK**	47	M	L	IIIB	3	2a		2		**>50%**
**OMCR16-014TK**	92	F	RT	IIA	3	0		2		
**OMCR16-016TK**	71	M	RT	IIIB	3	1a		2		
**OMCR16-017TK**	85	M	R	IIIB	3	2a		3	MSI	**>50%**
**OMCR16-021TK**	82	M	S	IIA	3	0		2		<50%
**OMCR16-027TK**	78	M	RT	IIIB	3	1a		2		
**OMCR16-030TK**	91	M	R	IIA	3	0		2	MSI	
**OMCR16-035TK**	56	M	RT	IIA	3	0		2		
**OMCR16-039TK**	73	M	T	IIIB	4a	1a		2		<50%
**OMCR16-047TK**	80	F	R	IIA	3	0		2		
**OMCR17-050TK**	82	M	R	IIIB	1	1a		2		<50%

Legend. Site: R = right; T = transverse; L = left; S = sigma; RT= rectum.

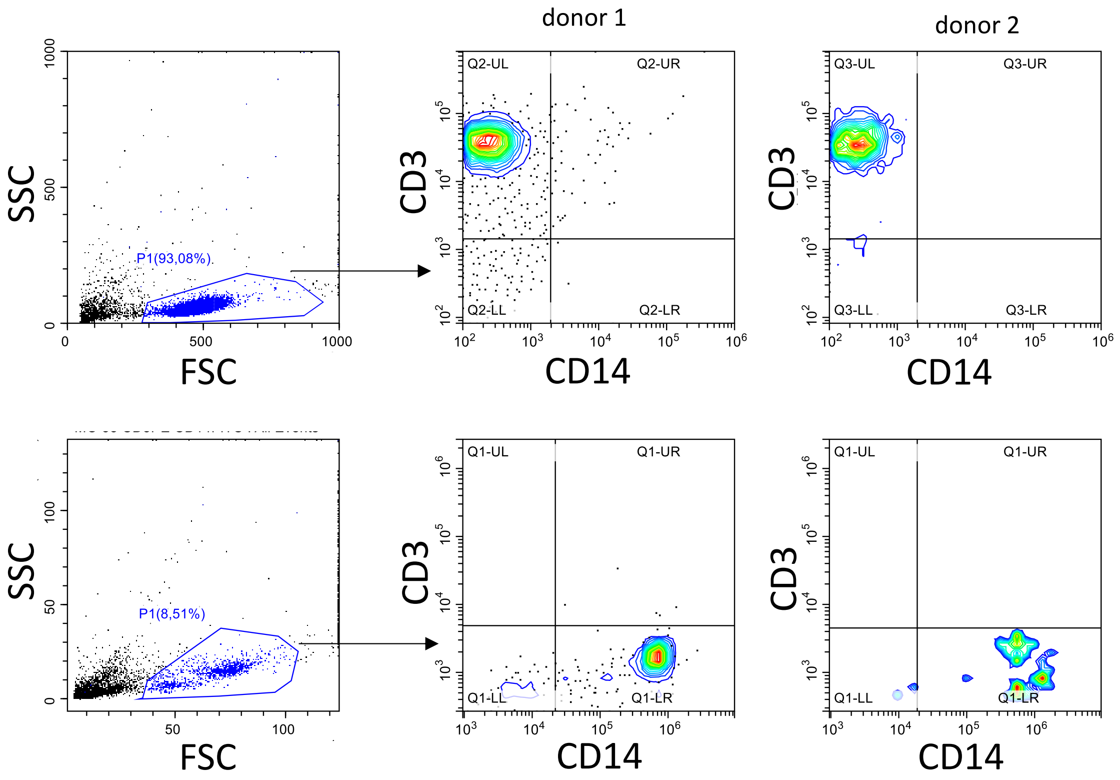

Figure S1. Degree of purity of T lymphocytes and monocytes isolated from PBMC. Purified T cells (upper plots) or monocytes (lower plots) were stained with anti-CD3 and anti-CD14 mAbs as indicated and analyzed with the Cytoflex S. Data are representative of two donors out of 10 tested. On the left the physical parameter SSC and FSC are shown. Plots show the expression of CD3 and/or CD14 antigens on gated viable cells identified in blue in the left plots.

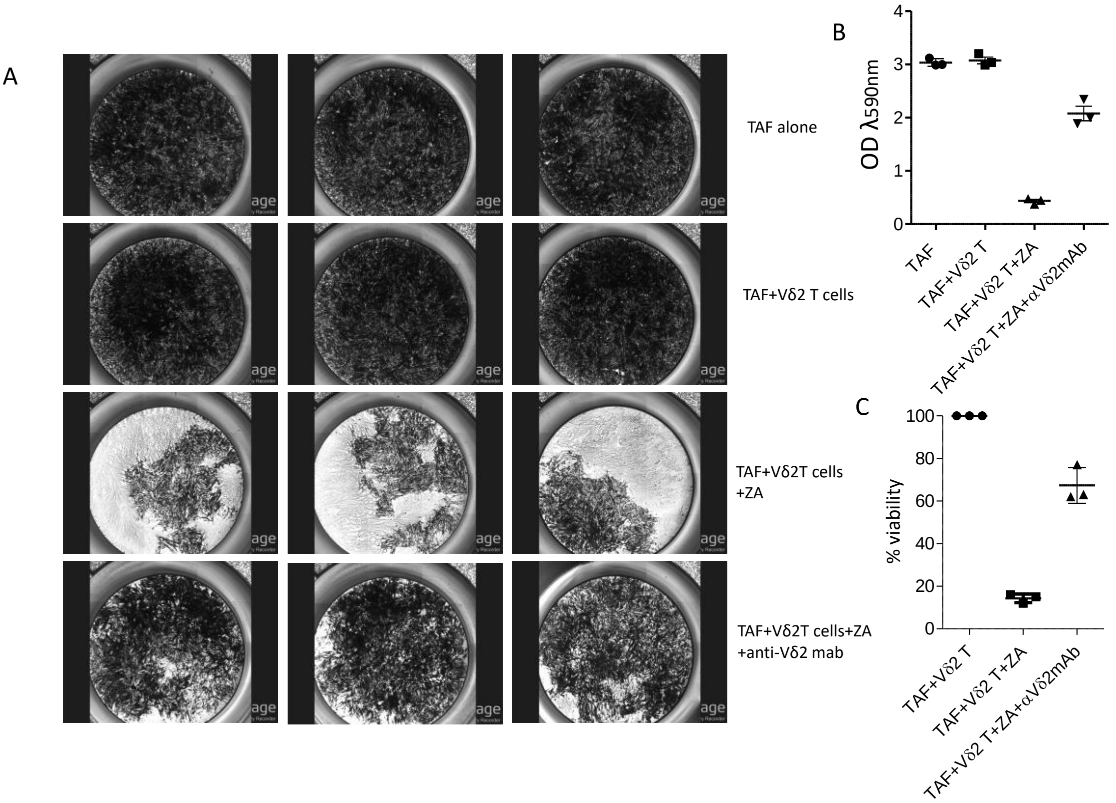

Figure S2. Evaluation of cytolytic activity of Vδ2 T cells with crystal violet assay. One representative experiment of the cell viability assay evaluated with crystal violet staining. A. TAF were seeded in flat-bottomed culture wells and stained with crystal violet either alone (first row) or in the presence of Vδ2 T cells, without (second row) or with 1.25μM of ZA (third row) and anti-Vδ2 mAb (fourth row). After staining with crystal violet (original color purple/blue, in images shown as black) some images of the whole wells were taken with the July-Stage microscope scanner with a 4x objective (NanoEnTek Inc, Seoul, Korea). The same experimental conditions were replicated at least in 3 different wells as shown. After elution of the crystal violet, the OD were evaluated at the wavelength of 590nm with the VICTOR X5 and plotted as shown in panel B. C. The percentage of viability was calculated considering as 100% the OD at 590nm of target cells alone (OD_{CTR}, in this case for TAF) compared with the OD at 590nm of the other experimental conditions according to the formula: OD_{exp}/OD_{CTR} x100= % viability.

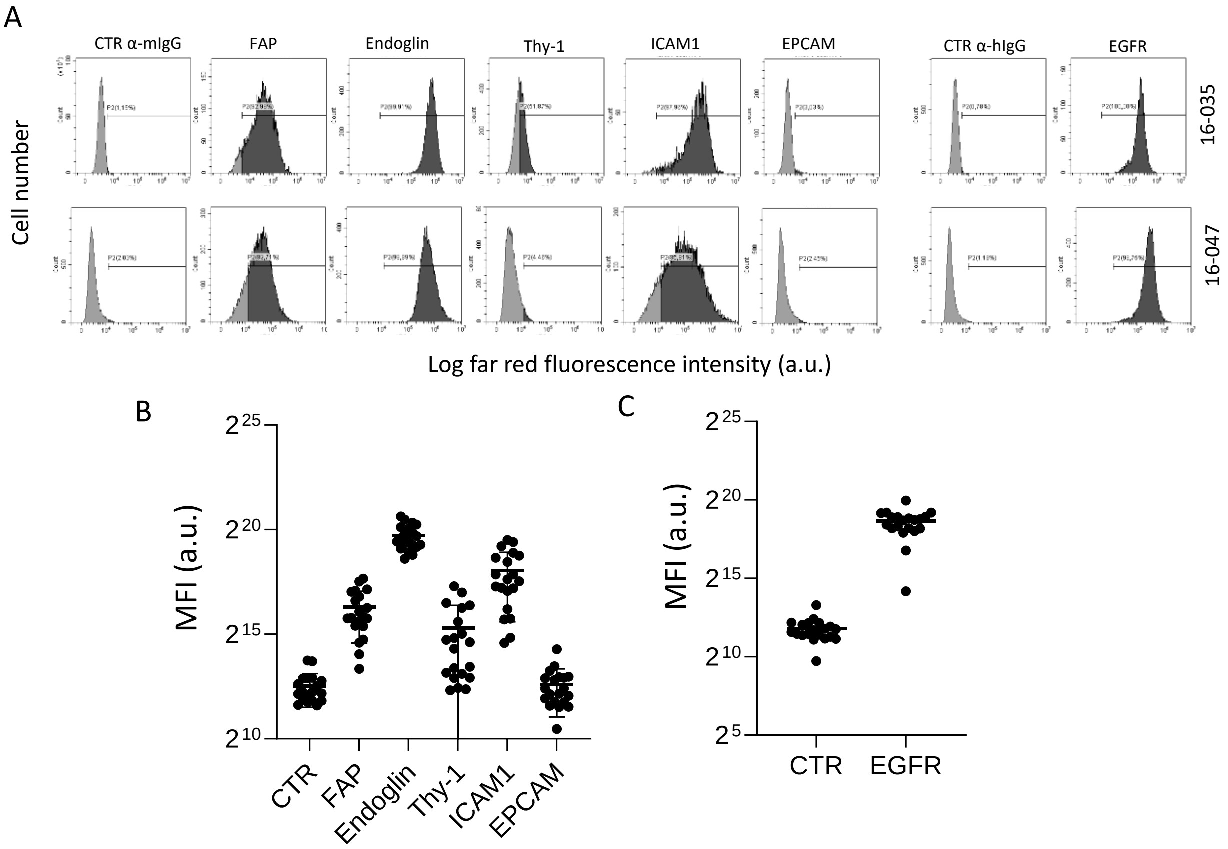

Figure S3. Phenotype of CRC-TAF. A: CRC-TAF phenotype (two representative cases, 16-035, 16-047) performed with the mAbs to the indicated molecules. Control samples were stained with isotype-matched irrelevant mAb (CTR α-mIg or CTR α-hIg). The anti-EGFR Cet was used, followed by APC-labelled anti-human Ig (α-hIg) antiserum. Results are expressed as Log red fluorescence intensity vs. number of cells. B: CRC-TAF phenotype (20 cases) performed and analyzed as in panel A; results are expressed as mean fluorescence intensity (MFI, a.u.). CTR: CTR-α-mIg. C: EGFR expression on CRC-TAF (20 cases) analyzed as in panel A and shown as MFI, a.u. CTR: APC-α-hIg.

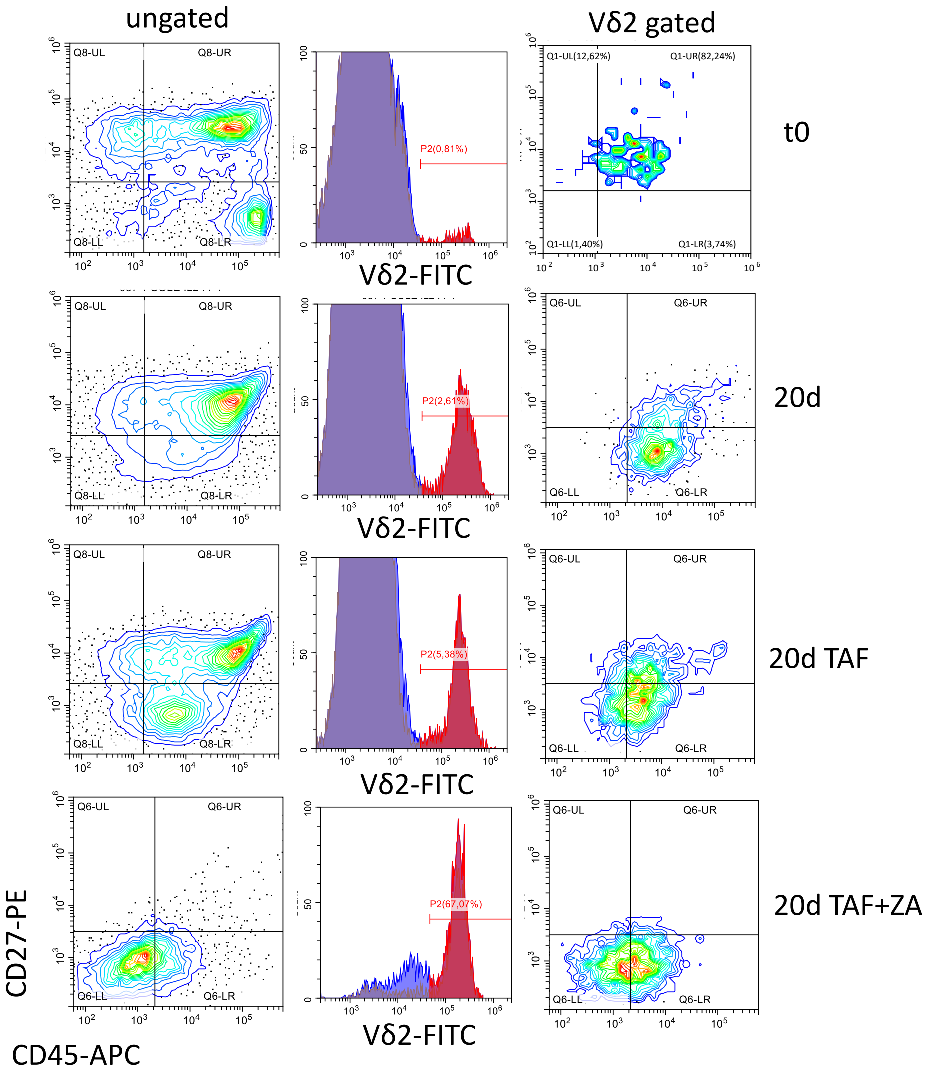

Figure S4. Gating strategy to analyze CD45RA and CD27 on Vδ2 T cells. T cells at the onset of cell culture (t0) or after 20d either alone (20d) on with untreated (20d TAF) or ZA-treated (20d TAF+ZA) CRC-TAF (16-030) were labelled with the anti-CD45RA-APC and the anti-CD27-PE and the anti-Vδ2-FITC antibodies. The reactivity of anti-CD45RA and anti-CD27 mAbs on ungated cell cultures are shown on the left; cells reacting with anti-Vδ2-FITC mAb were gated (histograms in the central panels, P2 red gated cells) and analyzed for the expression of CD45RA and CD27 (right contour plots).

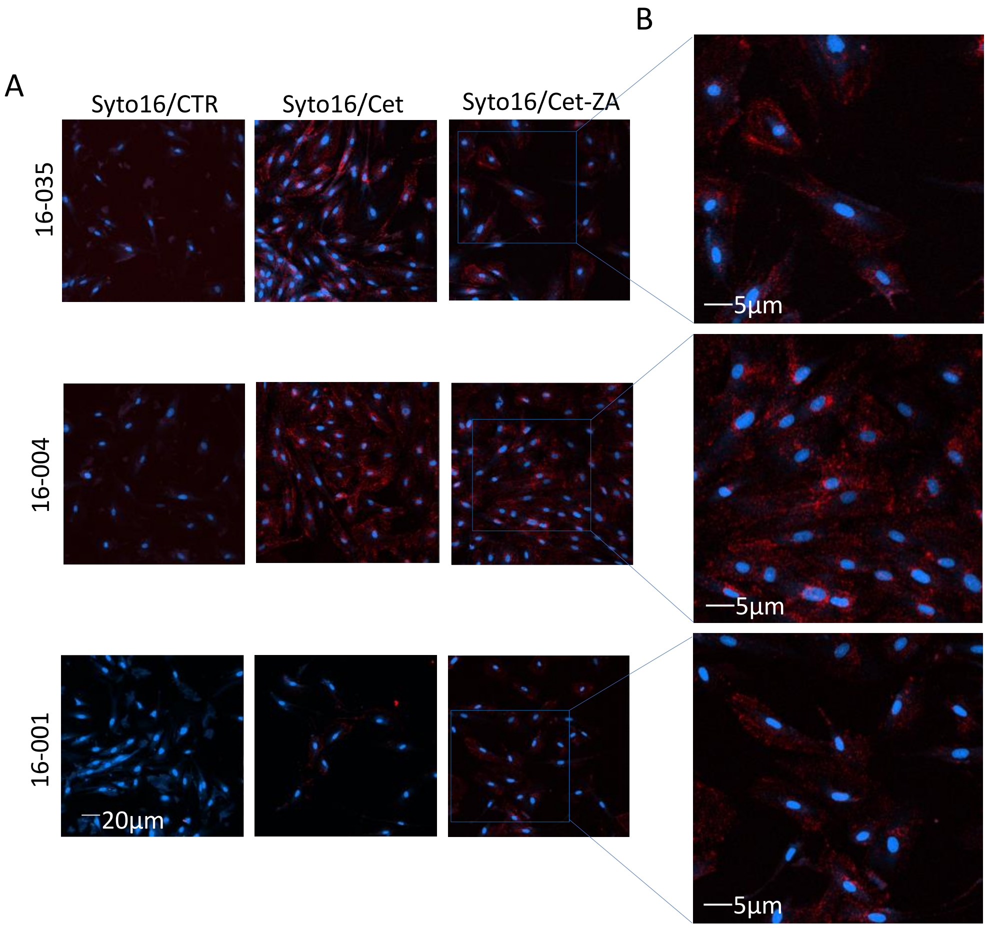

Figure S5. Confocal microscopy of Cet-ZA ADC reactivity with CRC-TAF. A: the three indicated CRC-TAF cell lines were stained with Syto 16 alone or together with 2.5µg/ml/10^6 cells of native Cet or Cet-ZA ADC followed by APC-anti-hIg antiserum. Samples were observed with the PlanApo 20X NA1.00 objective with the FV500 confocal Laser Scanning Microscope System Olympus. Image taken in sequence mode to avoid cross-talk between the fluorochromes and data analyzed with FluoView 4.3b computer software (Olympus). Results are shown in pseudocolor as surface cell membrane in red fluorescence vs nuclei in blue. B: enlargements of the squares depicted in the right images of panel A.
